# Modified posterior approach of the knee in patients with diffuse pigmented Villonodular synovitis: case series of a single Institution’s experience

**DOI:** 10.1186/s12891-022-05103-4

**Published:** 2022-03-03

**Authors:** Yi-Ping Wei, Shan-Wei Yang

**Affiliations:** Department of Orthopaedic, Kaohsiung veterans general hospital, 386, Ta-Chung 1st Rd, Kaohsiung, Taiwan, Republic of China

**Keywords:** PVNS, Posterior approach, Synovectomy, WOMAC, MSTS, Bone tumor

## Abstract

**Background:**

Diffuse pigmented villonodular synovitis (DPVNS) is a challenging tumor-like disorder that mainly occurs in the anterior aspect of the knee joint. The growth may sometimes extend to the posterior knee joint. Surgical excision is the mainstream treatment for DPVNS, and the posterior approach of tumor excision is adopted when the dominant tumor shows posterior extension. However, the optimal surgical approach over the posterior knee remains unknown.

**Methods:**

Patients with DPVNS of the knee joint who received the posterior approach of synovectomy from 1995 to 2019 were retrospectively reviewed to describe the modified separate posterior (SP) approaches, and evaluate the treatment outcomes in a case series of DPVNS knees. The results of the SP approach was also compared with those of traditional direct posterior (DP) approach. Postoperative functional outcomes were evaluated using the Western Ontario and McMaster Universities Osteoarthritis Index (WOMAC) standardized questionnaire and clinician-completed Musculoskeletal Tumor Society (MSTS) functional rating system at outpatient department.

**Results:**

A total of 20 DPVNS knees were included. Thirteen patients who received SP approaches were included in the SP group, while seven patients who received the DP approach were included in the DP group. The median follow-up times were 5.7 years (IQR, 2-8.8) in the SP group and 3 years (IQR, 2-5.3) in the DP group. Both groups showed satisfactory safety. The SP group presented higher postoperative mean WOMAC (91.23 ± 7.20) and mean MSTS (24.23 ± 2.68) than the DP group (mean WOMAC: 76.00 ± 16.57; mean MSTS: 22.43 ± 4.69). The Wilcoxon signed-rank test was use to compare preoperative and postoperative range of motion (ROM) for each group. The significant difference in SP group (*p* = 0.004) was found while *p* = 0.131 in DP group.

**Conclusions:**

The SP approach provides an effective approach with satisfactory outcomes for the surgical treatment of DPVNS knees.

## Introduction

Pigmented villonodular synovitis (PVNS) is a rare tumor-like disorder characterized by the clonal neoplastic proliferation of synovial-like mononuclear cells [1, 2]. These cells are admixed with multinucleated giant cells, foam cells, and inflammatory cells [3, 4]. The disorder causes progressive damage to the affected joint and may lead to arthritic degeneration and disability if left untreated [1, 5, 6]. Although PVNS is considered a benign tumor, it may be locally invasive with a tendency to recur; it may also be destructive and debilitating [7]. Research on the condition over the last decade has focused on how to eradicate PVNS [5, 8–10].

Two distinct types of PVNS have been identified: localized and diffuse (DPVNS); these types are histologically similar but differ in the extent of synovial involvement [2]. DPVNS is characterized by more marked symptoms, greater destruction, and higher recurrence rates compared with the localized type [11]. Surgical interventions include open surgery or arthroscopic excision; the mainstay treatment for tumor removal is complete surgical synovectomy [12]. However, complete tumor removal may be challenging in DPVNS. The recurrence rates observed after surgical resection range from 8 to 56% [13–15].

Adjuvant radiotherapy, including external beam radiation or intra-articular radioisotope injection-yttrium-90 or dysprosium-165, could be used to reduce recurrence [11, 16, 17]. Therapy targeting the CSF1 receptor, such as imatinib, nilotinib, emactuzumab, or PLX3397, is currently applied to control this disorder [18, 19].

Most cases of DPVNS are monoarticular, and the knee joint is the most frequently affected location [20, 21]. DPVNS mainly occurs in the anterior aspect of the knee joint but may sometimes extend to the posterior aspect of the joint [1]. In clinical practice, the posterior approach is less commonly used than the anterior approach. The combined posterior approach of synovectomy and tumor excision is adopted when the dominant tumor shows posterior extension [1, 2]. Direct posterior (DP) surgical dissection around the popliteal fossa of the knee joint is a traditional surgical approach of the posterior knee [22, 23]. As previously mentioned, the main limitation of DP approach is the risk to the neurovascular structures within the knee [23].

In this work, we report a modified posterior approach called the separate posterior (SP) approach on both sides of the knee joint for tumor excision of posterior DPVNS knees. This approach provides an easy means to expose the posterior capsule of the knee without direct dissection of the popliteal fossa and can avoid the risk of popliteal neurovascular injury. The purpose of this study is to describe this SP approach and evaluate the treatment outcomes of DPVNS knees. We also compared this technique with the traditional DP approach.

## Methods

### Study design and populations

This research represents a retrospective case series in a level 1 medical center. After receiving Institutional Review Board approval, we searched the pathology database of our institute for patients who received knee synovectomy for the pathologic diagnosis of PVNS from 1995 to 2019. Those who had diffuse PVNS of the knee joint and received the posterior approach of synovectomy were enrolled to the study. PVNS originating from joints other than the knee and localized PVNS were excluded. Patients with previous fracture or previous cruciate ligament injury in the operative knee joint were also excluded. Clinical data were retrospectively collected from medical records, imaging studies, and surgery notes. Subjects were included if they were over 18 years of age. The minimum followup was 2 years.

Localized or diffuse PVNS was determined by preoperative magnetic resonance imaging (MRI) or operative findings. The major feature of localized PVNS is localized nodules with a clear boundary. In diffuse PVNS, the surface of the synovial membrane is hemosiderin-villous, and extensive involvement of the adjacent soft tissue may be observed.

The data collected included the patients’ age, gender, treatment modalities, acceptance of adjuvant radiation treatment, and current disease status from medical records, image and telephone visit. All of the patients included in this work were divided into two groups. Patients who received the SP approach were included in the SP group, while those who received the traditional DP S-curved approach were included in the DP group. Patients in the SP group were operated on by one orthopedic surgeon, while patients of the DP group were operated on by two other orthopedic surgeons. The anterior incision of both groups was performed by the medial parapatellar midline approach.

### Operative technique and interventions

During this period, we treated all patients meeting the criteria in the same manner. Preoperative MRI was routinely performed on all patients for preoperative planning. Distribution of the disease was determined by T2-weighted images. The patients were assessed to confirm the location of the tumor and whether it was anterior or posterior dominant and lateral or medial dominant. The choice of approach depended on the distribution of PVNS in the knee joint. If the tumor involved the anterior aspect of the knee joint, the anterior midline longitudinal skin incision and standard medial parapatellar arthrotomy was performed. The anterior synovectomy was performed including the medial gutter, lateral gutter, intercondylar notch and suprapatellar pouch with a closed drain placement. If the tumor extended to the posterior aspect of the knee joint, the posterior approach was selected simultaneously.

**Fig. 1:**
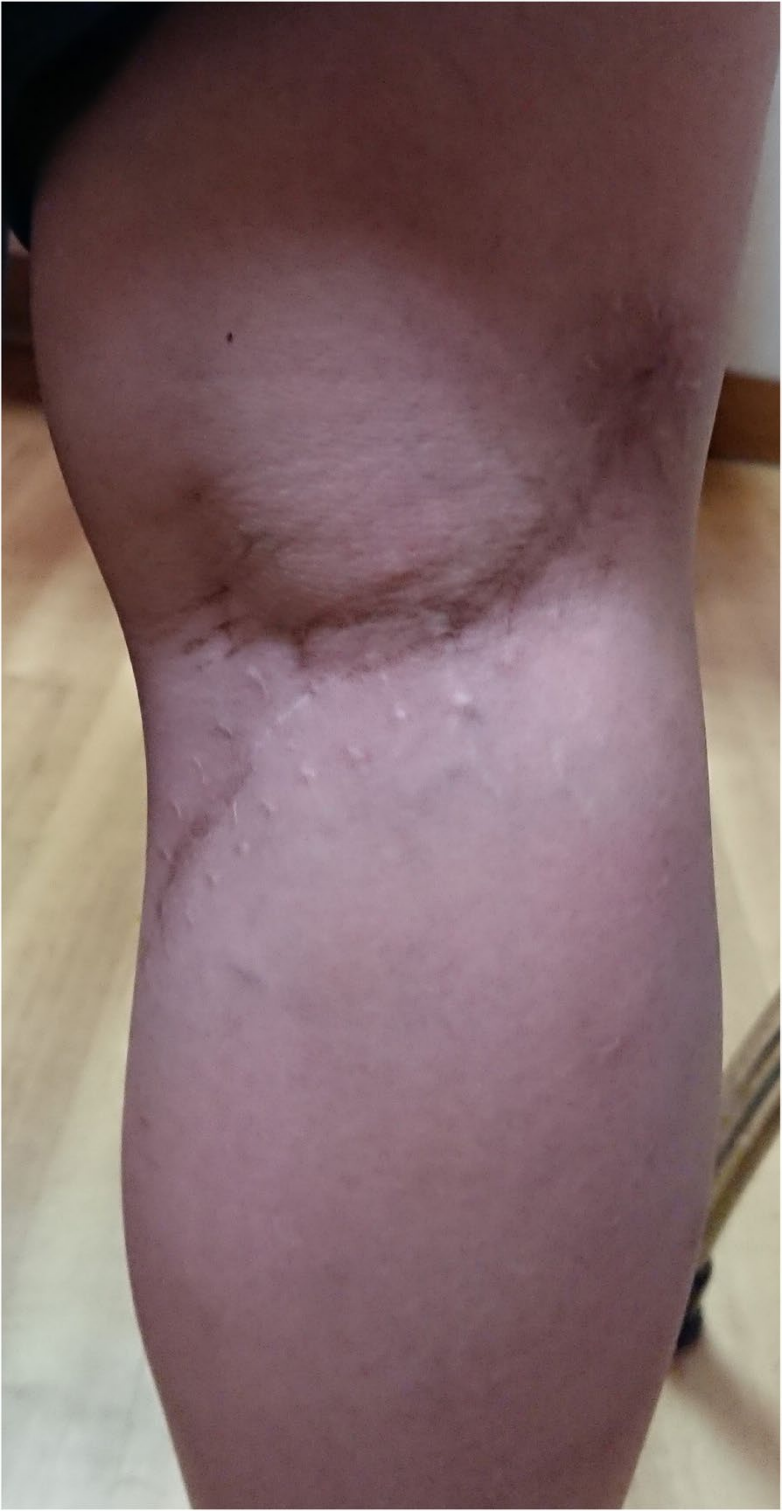
Postoperative image of the traditional DP S-curved incision in our case

The traditional DP S-curved incision was performed over the popliteal region to dissect the neurovascular bundle directly and expose the posterior capsule of the knee. (Fig. 1.) Tumors were removed after opening the posterior capsule. Three patients in DP group were treated by SWY, and four patients were treated by YCL.

**Fig. 2:**
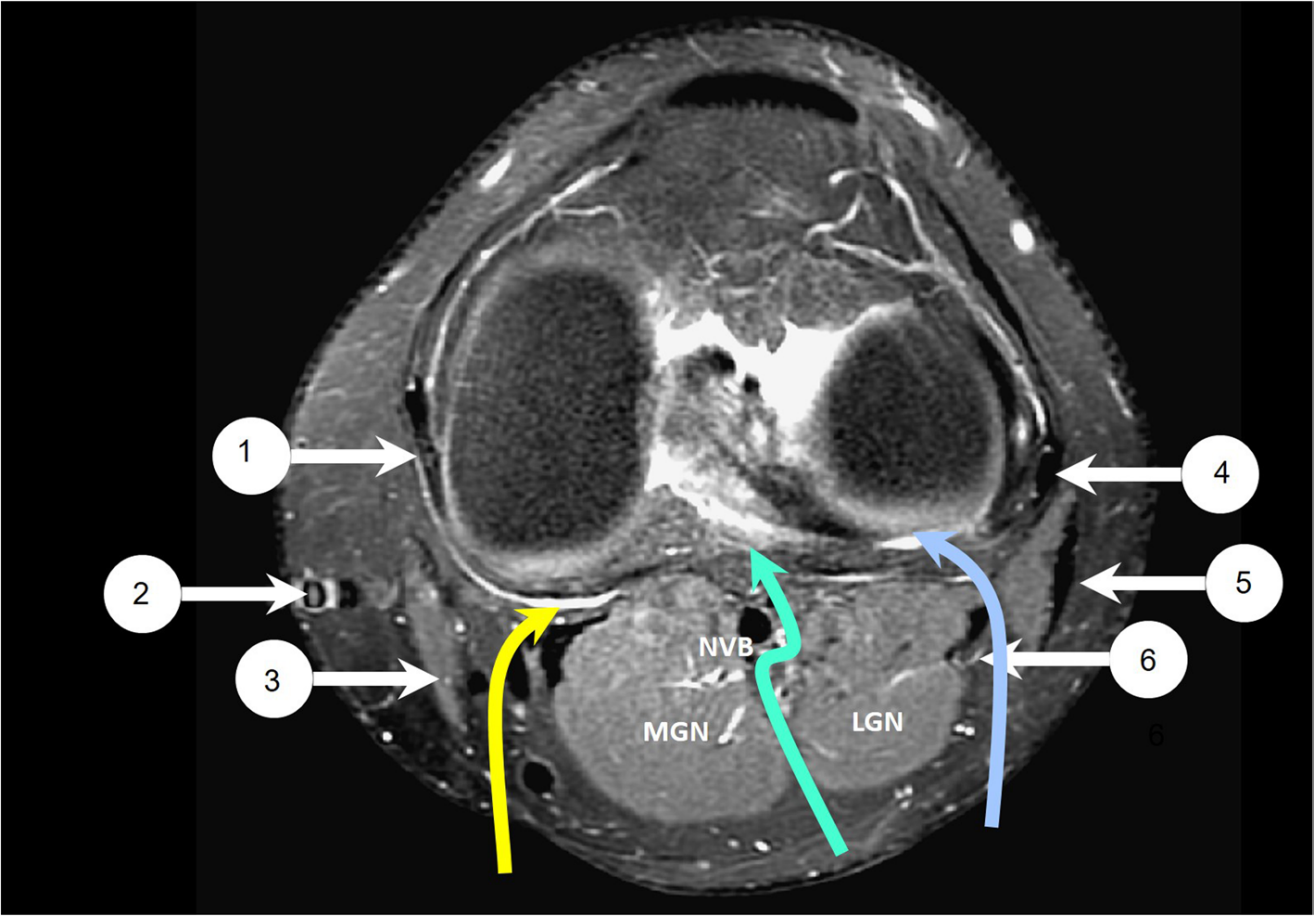
Left knee MRI of a healthy 21-year-old man. *Proton density* with fat saturation sequences axial view 1. Medial collateral ligament; 2. Great saphenous vein; 3. Sartorius and gracilis muscle; 4. Lateral collateral ligament; 5. Biceps femoris tendon and muscle; 6. Common peroneal nerve. Yellow arrow: PM approach; Green arrow: DP approach; Blue arrow: PL approach. MGN: Gastrocnemius muscle medial head; NVB: Neurovascular bundle; LGN: Gastrocnemius muscle lateral head.

**Fig. 3:**
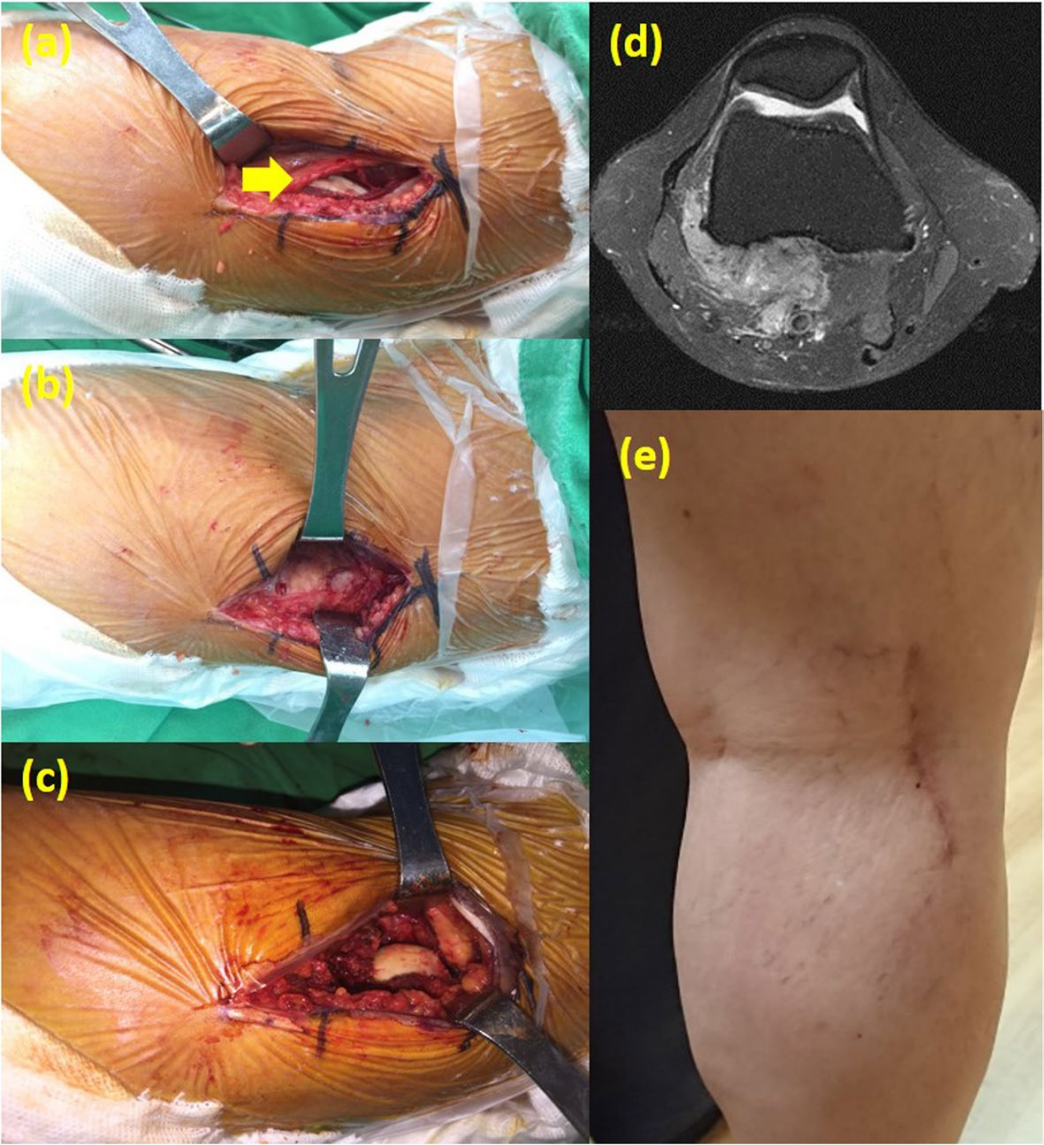
20-year-old man suffered from DPVNS of right knee. (a-c) PL approach. (a) Place the incision between the biceps tendon and lateral gastrocnemius. The common peroneal nerve lies in the lateral aspect of the popliteal space, medial to the biceps femoris tendon (see yellow arrow). **(b)** Lateral retraction of the biceps femoris protects the underlying peroneal nerve and exposes the posterolateral capsule.**(c**) After opening the capsule, lateral femoral condyle and PVNS tumor were seen deep to the joint capsule.**(d)** MRI of proton density with fat saturation sequences axial view showed the PVNS tumor located at anterior and posterior-lateral side. **(e)** Postoperative image of the operative knee with PL approach

**Fig. 4:**
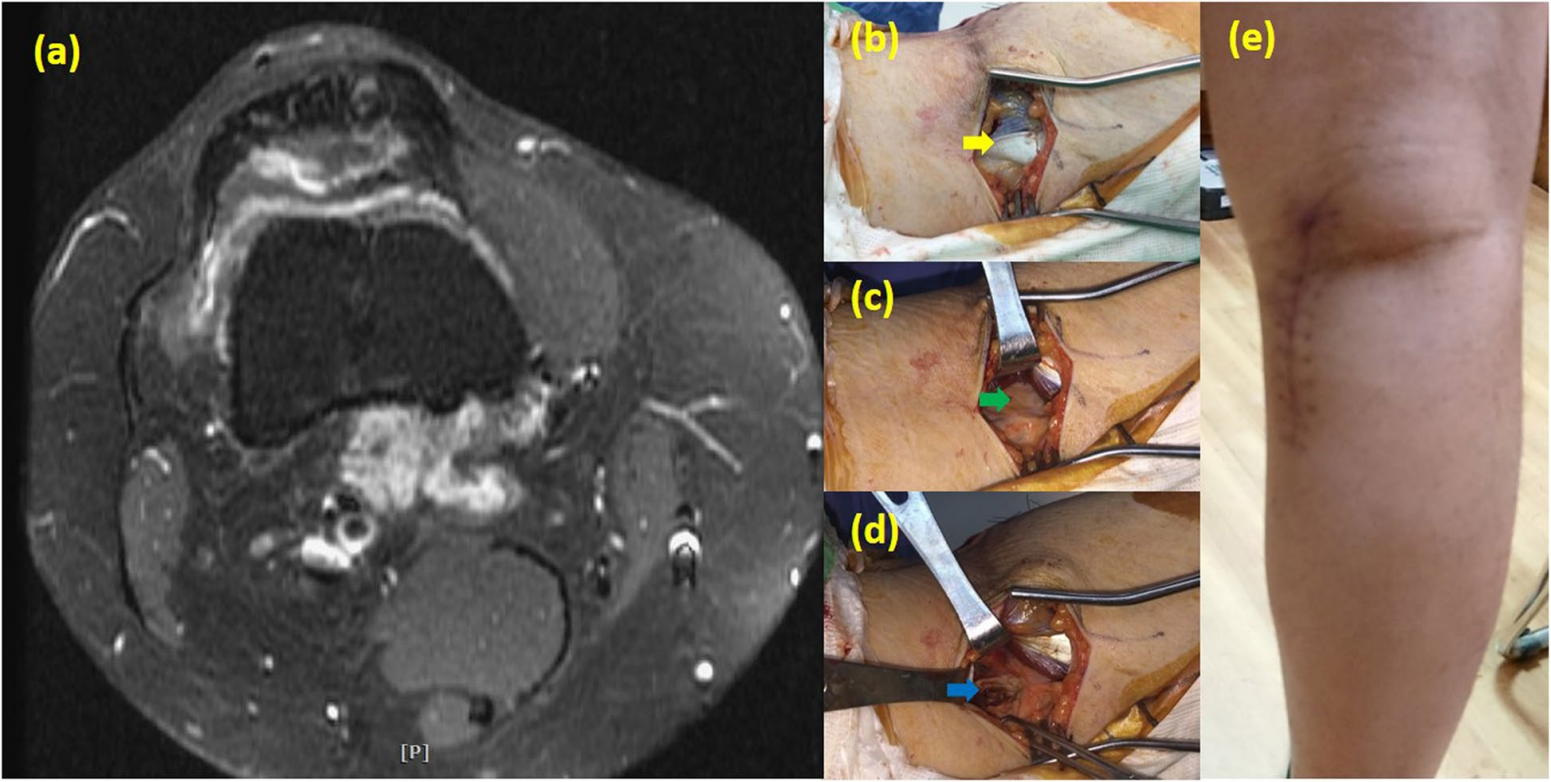
44-year-old woman suffered from DPVNS of right knee. (b-d) PM approach. **(a)** MRI of proton density with fat saturation sequences axial view showed the PVNS tumor located at anterior and posterior-medial side. **(b)** Place the incision over the medial head of the gastrocnemius. Yellow arrow: Gastrocnemius muscle medial head. **(c)** After retracting the medial gastrocnemius medially, the posterior-medial capsule was exposed (see green arrow). **(d)** After opening the capsule, PVNS tumor were seen deep to the joint capsule. Blue arrow: PVNS tumor. **(e)** Postoperative image of the operative knee with PM approach.

SP incisions of the knee, including posterior medial (PM) and posterior lateral (PL) incisions, were performed over both sides of posterior knee along the medial and lateral gastrocnemius muscles. (Fig. 2.) After PL incision, the peroneal nerve was identified and protected, the lateral gastrocnemius muscle was retracted medially, and the posterior capsule was exposed. (Fig. 3.) After PM incision, the medial gastrocnemius muscle was retracted laterally, and the posterior capsule was exposed. (Fig. 4.) The tumor could be removed after opening the capsule from the bilateral posterior corner of the knee joint without dissection of the popliteal neurovascular bundle. All SP incisions were performed by one surgeon (SWY). Another drain tube was inserted around the posterior capsule after SP or DP incision.

If the tumor deviated to the lateral side and did not include the medial side, a single PL incision was applied. If the tumor was located at the medial side, PM incision was selected. If the tumor extended to both sides of the posterior knee joint, PM and PL incisions were simultaneously performed to remove the tumor. We had no absolute contraindications specifically related to the posterior approach of the knee.

Patients were encouraged to mobilize their diseased knee joint postoperatively. Full weight bearing was commenced as tolerated. Clinic visits were scheduled every 3 months for clinical follow-up to assess symptoms. MRI of the diseased knee was obtained every 6 months during the first 2 years and then annually thereafter.

### Outcome measurements

All of the patient data, including MRI reports, functional outcomes, operated knee active range of motion (ROM), and complications, were reviewed. We recorded the preoperative and postoperative maximum angles of knee extension and flexion. Detections of residual disease or recurrences were checked by the postoperative MRI image of the diseased knee. The operative notes were referenced to confirm which zone was free of tumor. And we also checked any visible tumor on the earliest postoperative MRI (6 months following the operation). The recurrence of the tumor was defined by comparing the size and location of tumor on subsequent images of MRI of different timing. If new tumor formation was seen on subsequent MRI, the recurrence of PVNS was confirmed. An experienced radiologist interpreted all MRI images for residual of recurrent PVNS. Functional outcomes were evaluated using the Western Ontario and McMaster Universities Osteoarthritis Index (WOMAC) standardized questionnaire and clinician-completed Musculoskeletal Tumor Society (MSTS) functional rating system at outpatient department or telephone visit when conducting the study by an independent orthopedic surgeon during 2019 to 2020. The WOMAC score is widely used to evaluate hip and knee osteoarthritis and other hip and knee disabilities. The WOMAC is a self-administered questionnaire consisting of 24 items divided into three subscales of pain, stiffness, and physical function. It is scored from 0 to 100, with higher scores denoting less perceived disability. The modified version (1993) of the MSTS rating system is a limb-specific measure composed of six items, including pain, function, emotion, support, walking, and gait. It is scored from 0 to 30, and higher scores denote less impairment.

### Statistical analysis

The primary objective of the present study is to compare outcomes between the DP and SP groups. The demographic characteristics between groups were analyzed with the Fisher’s exact test for categorical data, and with the Mann–Whitney U test for comparing the continuous data from the two groups. The Wilcoxon signed-rank test was used to compare paired data for each group. Variables were expressed as mean ± standard deviation (SD). Follow-up times for tumor recurrence status were expressed as median and interquartile range (IQR). Data were processed and analyzed using Statistical Package for the Social Sciences software (version 22.0, IBM Corp., Armonk, NY, USA). Differences were considered statistically significant when *p* < 0.05.

## Results

### Demographic characteristics

From 1995 to 2019, 22 consecutive cases with histologically confirmed DPVNS of the knee joint who received the posterior approach of synovectomy were reviewed. Of these 22 patients, one was excluded because she was younger than 18 years of age and one was excluded due to loss to follow-up. Finally, 20 patients were included in this study. (Table 1.)

**Table 1:** Characteristics of the study population

Case	Group	Age	Gender	Previous surgery	Incision	Perioperative RT	Follow-up (year)	Residual or recurrence	Preoperative ROM (degree)	Postoperative ROM (degree)
1	SP	44	M	–	Anterior+ combined PM and PL	+	2	–	0-105	0-120
2		37	F	–		–	8.5	Residual	0-90	0-135
3		24	M	–		+	5	Residual and recurrence	10-90	10-105
4		32	M	–		–	2	–	0-120	10-120
5		32	F	–		–	9.5	–	0-120	0-135
6		56	F	–		+	2	–	10-90	10-135
7		35	F	Excision (anterior)		+	4	–	0-120	0-135
8		20	F	–		+	2	–	0-120	0-135
9		77	M	–		–	5.7	–	0-120	0-135
10		50	M	–		+	9	–	0-100	0-100
11		44	F	R	Anterior+ PM	+	7	Recurrence	0-90	0-110
12		44	F	Excision (anterior)	PL only	–	9	Residual	0-120	0-120
13		20	M	–	Anterior+ PL	+	8.5	–	0-90	0-135
14	DP	58	M	–	Anterior+ DP	–	5.3	–	0-90	10-90
15		55	M	–		+	2	–	0-60	10-90
16		50	M	–		+	2	–	0-60	10-90
17		32	M	–		+	2	Residual and recurrence	0-135	0-135
18		45	M	–		+	4	–	0-120	0-120
19		60	F	–		+	3	Recurrence	0-90	0-90
20		21	M	–		+	11	–	0-100	0-120

Thirteen patients who received PM or PL incision or both were categorized into the SP group, and seven patients who received DP S-curved incision were categorized into the DP group. The median follow-up times were 5.7 years (IQR, 2-8.8) in the SP group and 3 years (IQR, 2-5.3) in the DP group. Eighteen of the 20 patients were primary disease, and two of the 20 patients had received prior synovectomy with anterior approach. These two patients were referred to our department for local recurrence of DPVNS at 1 and 1.5 years postoperatively, respectively. The prior surgeons performed only an anterior approach to excise the anterior lesion but left the posterior lesion.

With the MRI within 6 months following the operation, we confirmed residual PVNS in four of 20 patients. Of these four patients, three patients were in SP group, and one patient was in DP group. No patient had previous fracture or previous cruciate ligament injury in the operative knee joint.

No significant differences in age, gender, patient numbers with combined anterior incision, and patient numbers with combined adjuvant radiotherapy were noted between the two groups (Table 2).

**Table 2:** Group comparison

Characteristics	SP group (***n*** = 13)	DP group (***n*** = 7)	***p*** value
Age (years)	39.62 ± 15.78	45.86 ± 14.49	0.211 ^a^
Gender (male/female)	6/7	6/1	0.158 ^b^
Combined anterior incision (*n*)	12 (92.3%)	7 (100%)	1.000 ^b^
Combined adjuvant radiotherapy (*n*)	8 (61.5%)	6 (85.7%)	0.354 ^b^
Operation time (minutes)	115.00 ± 43.83 (PL only: 40; Anterior + PL: 100; Anterior + PM: 180; Anterior + PM + PL: 117.50 ± 37.88)	184.57 ± 68.59	0.019 ^a,c^
Blood loss (ml)	18.46 ± 11.97 (PL only: 5; Anterior + PL: 10; Anterior + PM: 20; Anterior + PM + PL: 20.50 ± 12.57)	47.14 ± 33.52	0.109 ^a,c^
Postoperative WOMAC	91.23 ± 7.20 (75–99)	76.00 ± 16.57 (48–98)	0.037 ^a^
Postoperative MSTS	24.23 ± 2.68 (20–28)	22.43 ± 4.69 (14–27)	0.485 ^a^
Recurrence (*n*)	2 (15.4%)	2 (28.6%)	0.587 ^b^
Preoperative active ROM of the knee (degree)	104.23 ± 16.56	93.57 ± 28.09	0.438 ^a^
Preoperative extension (degree)	1.54 ± 3.76	0 ± 0	0.588 ^a^
Preoperative flexion (degree)	105.77 ± 14.41	93.57 ± 28.09	0.351 ^a^
Postoperative active ROM of the knee (degree)	122.31 ± 14.52	100.71 ± 23.53	0.046 ^a^
Postoperative extension (degree)	2.31 ± 4.39	4.29 ± 5.35	0.485 ^a^
Postoperative flexion (degree)	121.15 ± 15.70	105.00 ± 19.37	0.081 ^a^
Postoperative ROM > 120° flexion (*n*)	8 (61.5%)	3 (42.9%)	0.642 ^b^

Continuous data (i.e., age, operation time, blood loss, WOMAC, MSTS, and ROM) are presented as mean ± SD (range); categorical data are presented as number (percentage)

^a^Mann–Whitney U test for comparing the continuous data from the two groups

^b^Fisher’s exact test for categorical data

^c^The operation time and blood loss was compared between 10 patients (who received anterior, PM and PL incisions in SP group) and 7 patients (who received anterior and DP incisions in DP group)

### Clinical outcomes

Postoperative WOMAC scores were higher in the SP group (91.23 ± 7.20) than in the DP group (76.00 ± 16.57), and the difference between groups was significant (*p* = 0.037). The mean MSTS of the two groups were 24.23 ± 2.68 and 22.43 ± 4.69 (*p* = 0.485). Among the patients in the SP group, ten received the anterior approach with both PM and PL incisions, and the mean WOMAC and MSTS scores of these patients were 89.40 ± 7.21 and 23.60 ± 2.59, respectively. One patient received PL incision only, and this patient’s WOMAC and MSTS scores were 95 and 24, respectively. Another one patient received the anterior approach with PM incision only, and this patient’s WOMAC and MSTS scores were 99 and 27, respectively. The last patient in the SP group received the anterior approach with PL incision only, and the WOMAC and MSTS scores of this patient were 98 and 28, respectively.

Of the four patients with postoperative residual tumor, the mean WOMAC and MSTS scores were 95.75 ± 2.87 and 25.00 ± 2.45.

Postoperative adjuvant radiotherapy was recommended for all diffuse PVNS. However, some patients refused radiotherapy because of consideration of malignant transformation. In our series, 14/20 (70%) patients received the adjuvant RT. The mean postoperative knee score for the 14 patients with perioperative radiotherapy was WOMAC: 86.43 ± 11.37and MSTS: 24.64 ± 2.85; the mean score for the other 6 patients was WOMAC: 84.67 ± 18.06 and MSTS: 21.17 ± 3.92.

At the knee joint, most functional activities require up to 120 degrees of knee flexion, rather than the full 135 degrees. And 9 of the 13 patients (69.2%) in the SP group could exceed a postoperative ROM of 120° knee flexion, only 3 of the 7 patients (42.9%) in the DP group could achieve the same. The ability to achieve the functional ROM differed between the two groups. (*p* = 0.356).

We compared the operation time and blood loss between 10 patients (who received anterior, PM and PL incisions in SP group) and 7 patients (who received anterior and DP incisions in DP group). Both the operation time and blood loss in SP group (115.00 ± 43.83 min; 18.46 ± 11.97 ml) were less than in DP group (184.57 ± 68.59 min; 47.14 ± 33.52 ml) (*p* = 0.019 and 0.109, respectively). However, the difference in the baseline incisions number between treatment groups weaken the clinical value of the observation.

Tumor recurrence postoperatively was higher in the DP group (28.57% in median follow-up 3 years) than in the SP group (15.38% in median follow-up 5.7 years) without significant difference (*p* = 0.587). The most common complaints of the patients after surgery were joint effusion and swelling sensation. Among the 20 patients enrolled in this study, 2 cases in the SP group and 2 cases in the DP group reported these complaints during follow-up. Symptoms of discomfort gradually improved over the course of follow-up. Complications such as neurovascular bundle injury, paresthesia, dysesthesia, skin necrosis, and wound dehiscence were not observed in both groups.

## Discussion

Management of DPVNS involving the popliteal fossa remains a challenging endeavor [2]. Until now, the posterior open synovectomy has been proved to be the most direct and effective treatment for accessing posterior knee pathologies [23, 24]. The purpose of our study was to determine (1) local disease control, (2) the improvement of knee joint function, (3) whether there were any complications after SP incisions in patients with DPVNS.

In the past decade, numerous approaches to the posterior aspect of the knee have been described for fixation of posterior tibial plateau fractures [25, 26]. Luo et al., for instance, popularized the L-shaped incision, which was originally described by Lobenhoffer [27, 28]. Since then, the L-shaped incision is commonly used to manage posterior column tibial plateau fractures; however, complications associated with raising a fascial flap can occur. In a cohort of 12 patients, Bhattacharyya et al. used the DP approach for the open reduction and internal fixation of posterior tibial plateau fractures [26]. The exposure provided by the DP approach facilitates anatomic fracture reduction, but it requires wide medial-to-lateral dissection. In this study, two cases presented postoperative complications: one wound dehiscence and one flexion contracture [26].

Berwin et al. described a safe posterior approach via a single longitudinal incision similar to that employed in the flexor carpi radialis approach for the wrist [29]. This incision is similar to our PM incision, and less dissection on neurovascular bundle was needed in this modified approach than in traditional DP approach [29].

Very few published clinical studies discussing the posterior approach for synovectomy or tumor excision, as well as the related recurrence rates and complications, are currently available [22, 24]. Ohnuma et al. reported five patients with DPVNS of the knee who underwent anterior and posterior synovectomy using two posterior oblique incisions [30]. Of the five patients, two patients experienced joint stiffness and received manual mobilization. Thus, knee function preservation may be a focus of attention after combined anterior and posterior synovectomy [30, 31].

In this study, we report a SP approach for DPVNS removal over the posterior knee. The results of our case series clearly showed good functional outcomes among our 13 patients receiving separate PM/PL or both incisions. Although both groups in our study showed satisfactory safety and high functional scores, the SP group presented better outcome scores and postoperative active ROM of the operated knee compared with the DP group. Among the 13 patients in the SP group, the mean maximal preoperative extension angle was 1.54 degrees, and developed to 2.31 degrees postoperatively. One patient (7.7%) had flexion contracture develop. The mean maximal preoperative flexion angle was 105.77 degrees and improved to 124.62 degrees postoperatively.

By comparison, among the seven patients with DP incision, the mean maximal preoperative extension angle was 0 degree, and developed to 4.29 degrees postoperatively. Three patients (42.9%) had flexion contracture develop. The mean maximal preoperative flexion angle was 93.57 degrees and improved to 105 degrees postoperatively.

The Wilcoxon signed-rank test for comparing preoperative and postoperative active ROM for each group, and showed *p* = 0.004 in SP group; *p* = 0.131 in DP group. The difference in outcomes between groups may be attributed to DP incision and popliteal dissection leading to contracture of posterior capsule and soft tissue scarring, which restricts the knee joint from achieving full extension [32]. Wu et al. [33] reported nine patients with DPVNS treating with staging anterior and DP approaches. Three patients (33.3%) experienced flexion contracture of 5 degrees develop postoperatively. Thus, flexion contracture of the knee may be a major concern after DP approach. SP incision on both sides of the knee can avoid direct popliteal dissection to prevent not only popliteal neurovascular bundle injury but also soft-tissue scarring, thus provides better postoperative ROMs and outcomes.

Prior to surgery, we assessed the location of the tumors by MRI. The PM and PL approaches can be used in combination or alone, depending on the location of the tumor. The SP approach on both sides of the knee allows for individual focused medial or lateral posterior exposure and avoids unnecessary wound extension and soft-tissue dissection [29, 32]. However, it is difficult to eradicate all DPVNS tissue, even using open synovectomy. So the residual tumor occupied large proportions in both groups (23.07% in SP and 14.29% in DP group). In our study, four patients had residual tumor after open synovectomy, including the two patients with subsequent local recurrence. None of these patients received additional surgery. The mean postoperative knee score for the four patients with residual PVNS (WOMAC 95.75 ± 2.87 and MSTS 25.00 ± 2.45) was not inferior than the mean score for the other 16 patients (WOMAC 83.44 ± 13.70; MSTS 23.25 ± 3.70) (*p* = 0.039 and 0.494, respectively).

The adjuvant radiotherapy was suggested for DPVNS [21]. We initiate radiotherapy at postoperative 4 weeks if no contraindications. Concerns regarding the open synovectomy following the adjuvant radiotherapy are joint stiffness and poor healing of wound. All the wounds healed well without superficial or deep infections in our case series. Additionally, the mean postoperative knee score for the 14 patients with perioperative radiotherapy (WOMAC 86.43 ± 11.37; MSTS 24.64 ± 2.85) was also not inferior than the mean score for the other 6 patients (WOMAC 84.67 ± 18.06; MSTS 21.17 ± 3.92) (*p* = 0.968 and 0.041, respectively) following our protocol for postoperative rehabilitation (immediate weight bearing and mobilize the knee without limited ROM).

Accurate delineation of the relevant knee anatomy is essential in efforts to design the optimal surgical approach. The most important structures to consider include the popliteal artery, popliteal vein, and tibial nerve [22]. Previous researchers demonstrated that the safe distance to the popliteal vasculature is, on average, 4.27 ± 0.05 mm posterior to the joint capsule at the level of the medial epicondyle, as measured in 12 cadaveric male knees with an age range of 68–81 years [34]. The DP approaches, especially directly dissecting the intercruciate region and posterior joint capsule, may injure popliteal neurovascular bundle.

In our study, four of the twenty patients present disease recurrence (20%; 15.4% in SP group and 28.6% in DP group). This was close to the recurrence rate (11-20%) described in previous studies of DPVNS treated with anterior and posterior synovectomy [30, 31, 33, 35]. (Table 3).

**Table 3:** Comparison of literature reporting results for DPVNS of the knee

Study	Incisions	Patient number	Follow-up	Perioperative radiotherapy	Residual tumor or recurrence	Knee ROM	Postoperative flexion contracture develop	Complication
Ohnuma et al. 2003	Anterior and two posterior oblique incisions (2-stage)	5	Mean 6.9 years (3.3-8.1 years)	No	One residual (20%); one recurrence (20%)	Three (60%) full ROM	Not available	One infection
Flandry et al. 1994	Anterior and one posteromedial incisions (2-stage)	23	Mean 58 months	No	One recurrence (11%)	Not available	Not available	Not available
Wu et al. 2007	Anterior and direct posterior incisions (2-stage)	9	Mean 67 months (37-103 months)	Yes	Two recurrence (8%)	Maximal flexion from 90 to 130 degrees.	Three (33.3%) had flexion contracture of 5 degrees develop.	None
Chen et al. 2012	Anterior and direct posterior incisions	19	Median 98 months (42-130 months)	Yes	Five residual (26%); two recurrence (11%)	Mean extension improved from 11 to 2 degrees; flexion from 76 to 127 degrees.	None	None
Current study	1. Anterior and two separate posterior incisions 2. Anterior and direct posterior incisions	20	Median 4.5 years (2-11 years)	14 patients with perioperative radiotherapy (70%)	1. SP group: 3 residual (23.1%) and 2 recurrence (15.4%) 2. DP group: 1 residual (14.3%) and 2 recurrence (28.6%)	1. SP group: mean extension from 1.54 to 2.31 degrees; flexion from 105.77 to 124.62 degrees. 2. DP group: mean extension from 0 to 4.29 degrees; flexion from 93.57 to 105 degrees.	1. SP group: One (7.7%) had flexion contracture of 10 degrees develop. 2. DP group: Three (42.9%) had flexion contracture of 10 degrees develop.	None

Our study presents some limitations. First, the sample size is quite small because of the rarity of PVNS; thus, the statistical power of our results may be compromised. Second, decisions related to the surgical modality to apply and radiotherapy use may cause selection bias in retrospective observational studies. Third, the follow-up times differed among the 20 patients. Fourth, the disease courses among the 20 patients differed. Two had received synovectomy before our procedure and this might have affected the surgical outcome. Fifth, data of the preoperative functional score in our patients is not complete. Thus, further research with a larger cohort is warranted to confirm our findings.

## Conclusion

Separate PM/PL or both incisions and the traditional DP S-curved incision of the knee can provide effective approach with satisfactory outcomes in the treatment of PVNS knees. However, the posterior approach of separate PM/PL or both appears to be associated with better postoperative functional outcomes and active ROM of operative knees than the traditional DP approach.

## Data Availability

The dataset(s) supporting the conclusions of this article is (are) included within the article.

## Abbreviation

DPVNS: Diffuse pigmented villonodular synovitis
SP: Separate posterior
PM: Posterior medial
PL: Posterior lateral
DP): Direct posterior
WOMAC: Western Ontario and McMaster Universities Osteoarthritis Index
MSTS: Musculoskeletal Tumor Society functional rating systems
MRI: Magnetic resonance imaging
ROM: Range of motion
IQR: Interquartile range
