# Correction: Modified posterior approach of the knee in patients with diffuse pigmented Villonodular synovitis: case series of a single Institution’s experience

**DOI:** 10.1186/s12891-022-05459-7

**Published:** 2022-06-06

**Authors:** Yi-Ping Wei, Shan-Wei Yang

**Affiliations:** Department of Orthopaedic, Kaohsiung Veterans General Hospital, 386, Ta-Chung 1st Rd, Kaohsiung, Taiwan, Republic of China

**Correction: BMC Musculoskelet Disord 23, 197 (2022)**

**https://doi.org/10.1186/s12891-022-05103-4**

Following the publication of the original article [1] the authors reported that a certain part of Table 2 has been mistyped.

The original article [1] has been updated.

See page 2 for the corrected part of the table.

**Table 2:**
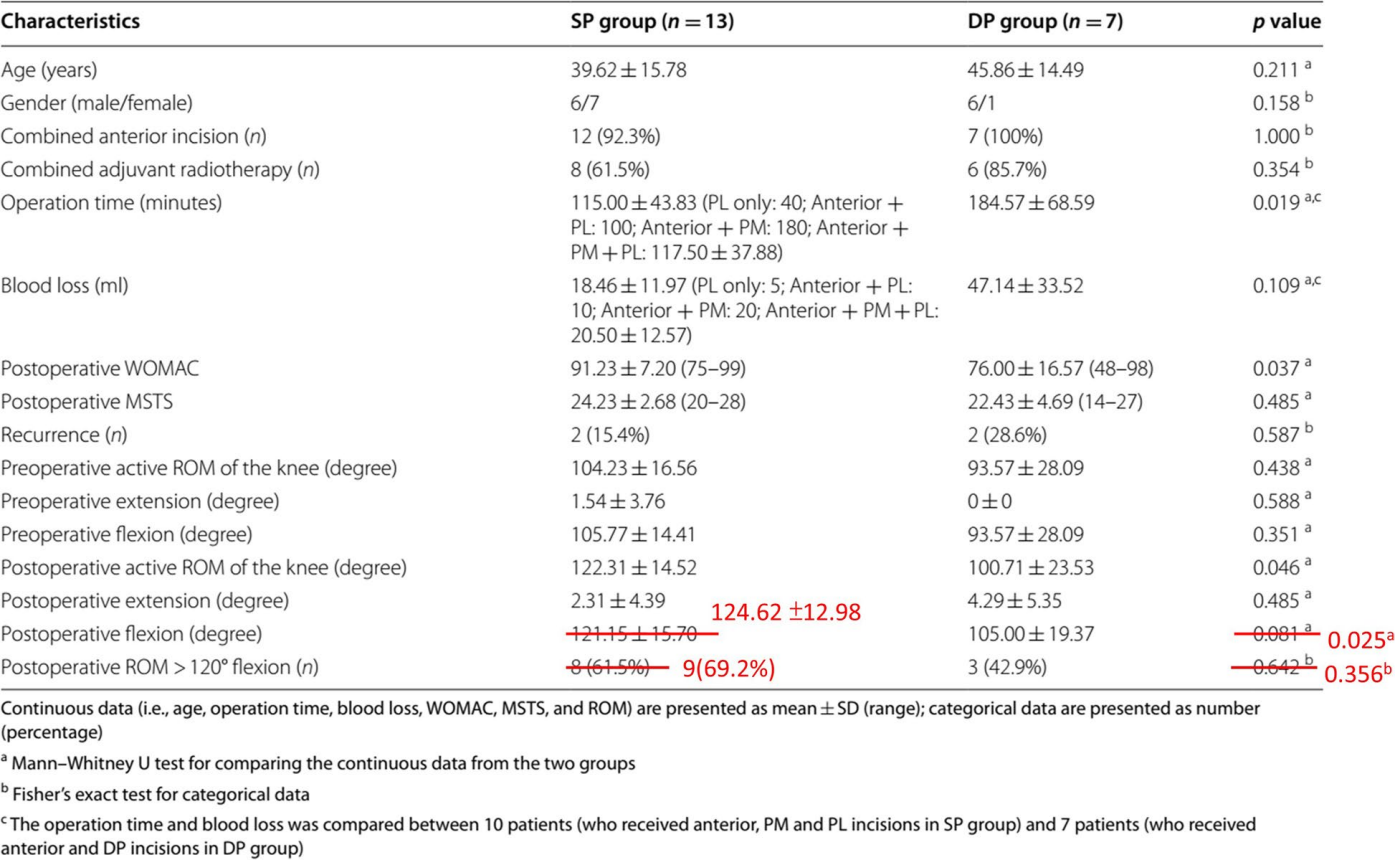
Group comparison
